# Adipocytes *ESR1* Expression, Body Fat and Response to Testosterone Therapy in Hypogonadal Men Vary According to Estradiol Levels

**DOI:** 10.3390/nu10091226

**Published:** 2018-09-04

**Authors:** Georgia Colleluori, Lina E. Aguirre, Clifford Qualls, Rui Chen, Nicola Napoli, Dennis T. Villareal, Reina Armamento-Villareal

**Affiliations:** 1 Department of Medicine, Baylor College of Medicine, One Baylor Plaza, Houston, TX 77030, USA; colleluo@bcm.edu (G.C.); Rui.Chen2@bcm.edu (R.C.); dennis.villareal@bcm.edu (D.T.V.); 2 Center for Translational Research on Inflammatory Diseases, Michael E. DeBakey VA Medical Center, 2002 Holcombe Blvd, Houston, TX 77030, USA; 3 Department of Internal Medicine, University of New Mexico, Albuquerque, NM 87131, USA; aguirre.lina1@gmail.com; 4 New Mexico VA Health Care System, 1501 San Pedro SE, Albuquerque, NM 87108, USA; 5 Division of Mathematics and Statistics, University of New Mexico School of Medicine, Albuquerque, NM 87108, USA; CQualls@salud.unm.edu; 6 Department of Endocrinology and Metabolism, University Campus Biomedico of Rome, Via Alvaro del Portillo 21040, 00128 Rome, Italy; n.napoli@unicampus.it

**Keywords:** oestrogen receptor α, testosterone, estradiol, fat mass, obesity, hypogonadism

## Abstract

Estradiol (E2), mainly produced from Testosterone (T) in men, promotes visceral lipolysis. However, high visceral fat and hyperestrogenemia are features of obese hypogonadal (HG) men. Our study objectives are to evaluate relationships between circulating E2 and: (1) fat mass; (2) Estrogen Receptor α (*ESR1)* expression in subcutaneous adipose tissue; (3) changes in body fat after 6 months (M) of T therapy in HG men. Hypotheses: (1) existence of a range of circulating E2 associated with better body composition; (2) serum E2 determines tissue E2 sensitivity which affects response to T therapy. Men 40–74 years old, T < 300 (ng/dL), given T-cypionate for 6 months. Subjects were divided into 4-E2 categories: (1) <10.0; (2) 10.0–15.9; (3) 16.0–19.9; (4) ≥20.0 (pg/mL). Body composition (DXA), fat biopsies (liposuction), gene expression (qPCR), serum E2 and T (LC/MS), at baseline and 6 months. We enrolled 105 men; 90 completed the study. Group 2 had lower total and truncal fat mass (*p* < 0.01) but higher % lean mass (*p* < 0.001). *ESR1* mRNA was the highest in group 1 (*p* = 0.01). At 6 months, group 1 had higher reduction in total (*p* = 0.03) and truncal (*p* = 0.01) fat. In conclusion, serum E2 = 10–15.9 (pg/mL) is associated with the best body composition profile in HG men; however, those with E2 < 10 (pg/mL) had the best response (greater fat loss) to T replacement possibly because of greater E2 sensitivity.

## 1. Introduction

Obesity and its related comorbidities are leading causes of morbidity and mortality worldwide [1]. High visceral fat mass is a risk factor for the development of cardiometabolic diseases [1,2], reason for which research investigating mediators of adipose tissue deposition and distribution is critical. Oestrogen stimulates visceral lipolysis and subcutaneous adipogenesis, promoting the development of a gynoid, as opposed to an android, body habitus [3,4,5,6]. Importantly, oestrogen’s role as fat mass regulator is not limited to females. Contrary to past belief, oestrogen and not testosterone (T), is the main sex hormone regulating body fat in men [7]. Although low circulating T results in high fat mass, recently, this phenotype is no longer attributed to the lack of T action but to the reduced amount of T available for conversion into oestrogen by the aromatase enzyme [7]. Consistently, men with aromatase deficiency, who have severe oestrogen reduction but normal testosterone, have truncal obesity, while treatment with estradiol (E2) results in reduction of truncal fat [8]. On the other hand, high visceral fat mass and hyperestrogenemia are features of obese individuals, which seem counterintuitive considering oestrogen’s effects on adipose tissue. It is thus possible that obesity-induced hyperestrogenemia results in reduced oestrogen sensitivity. We recently reported that fat mass in postmenopausal women follows a U-shaped trend based on estradiol levels, with individuals belonging to the lowest and highest E2 quartiles having greater fat mass [9]. We speculated that the increased fat mass at the higher end of the E2 spectrum could be due to resistance to E2 action, similar to the findings observed in oestrogen receptor α (*ESR1*) deficient humans [8,10].

The objectives of this study were to evaluate the relationship between circulating E2 and: (1) fat mass; (2) *ESR1* expression in the subcutaneous adipose tissue; (3) changes in body fat after 6 months of T therapy in hypogonadal (HG) men. We hypothesize the existence of a range of circulating E2 associated with better body composition and that serum E2 determines tissue E2 sensitivity which affects response to T therapy in HG men. In this study, we reported that: (1) fat mass follows a U-shaped distribution based on E2 levels and circulating E2 between 10.0 and 15.9 (pg/mL) is associated with better body composition profile in HG men; (2) HG men with lower serum E2 have higher subcutaneous adipocyte *ESR1* expression and lose more fat mass in response to 6 months of T therapy. 

## 2. Materials and Methods 

### 2.1. Study Design and Study Population

This study includes 105 subjects who participated in a single-arm clinical trial (gov Identifier: NCT01378299) investigating the pharmacogenetics of response to T therapy in HG men [11,12]. This particular report consists of the data collected in the first 6 months of the study. Hypogonadism in men was defined as an average total T of <300 (ng/dL) (10.41 nmol/L) from 2 samples collected between 8:00 and 11:00 a.m., 30 min apart at least [13]. The protocol was approved by University of New Mexico and Baylor College of Medicine Institutional Review Boards. The study was conducted at the New Mexico VA Health Care System (NMVAHCS, IRB: 11.139) and at the Michael E. DeBakey VA Medical Center (MEDVAMC, IRB: H-34812) in accordance to the guidelines in the Declaration of Helsinki for the ethical treatment of human subjects. Participants were recruited by flyers and postcards to patients attending the Endocrine, Urology and Primary Care Clinics of the New Mexico VA Health Care System and Michael E. DeBakey VA Medical Center, or letters sent to primary care physicians. All subjects signed a written informed consent before study participation. Inclusion criteria: male veterans between 40–74 years of age with no medical problems that may prevent them from finishing the study. Exclusion criteria: treatment with bone-acting drugs (e.g., bisphosphonates, selective oestrogen receptor modulators, glucocorticoids, androgen deprivation therapy, sex-steroid compounds and anticonvulsants); therapy with finasteride; osteoporosis; history of fragility fractures; diseases known to affect bone metabolism such as: hyperparathyroidism, chronic liver disease, uncontrolled or untreated hyperthyroidism, significant renal impairment, history of prostate cancer and breast cancer; and untreated sleep apnea.

### 2.2. Testosterone Therapy 

All participants received testosterone cypionate injections intramuscularly. The starting dose was 200 mg every 2 weeks and was adjusted to target serum T level between 500–800 (ng/dL). Although T dose was adjusted to target a serum T level of 300–600 (ng/dL) upon the direction of the Food and Drug Administration in the latter part of the study, the subjects who had a fat biopsy at 6 months were not affected by this change. Dosage adjustments were also made based on haematocrit levels. 

### 2.3. Biochemical Data

Screening T was measured by immunoassay at the NMVAHCS and MEDVAMC clinical laboratories. Serum samples were collected at baseline and after 6 months of intervention and were used to assess E2 and T by liquid chromatography/mass spectrometry (LC/MS), (analytical measurement range: 0.3–200 (pg/mL), Mayo Clinic Laboratories, Rochester, MN, USA) at the end of the trial.

### 2.4. Body Mass Index and Body Composition 

Body weight and height were measured by standard weighing scale and stadiometer, respectively. Body Mass Index (BMI) was calculated as body weight in kg divided by the square of the height in meters (kg/m^2^). Body composition assessment was performed by dual-energy X-ray absorptiometry (Hologic-Discovery; Hologic Inc, Bedford, MA, USA; Enhanced Whole Body 11.2 software version; Hologic) as previously described [14]. Images were analysed, following the manufacturer’s instructions. The coefficient of variability (CV) for lean and fat mass assessment in our laboratory is 1.5% [14]. BMI and body composition were assessed at baseline and 6 months.

### 2.5. Subcutaneous Adipose Tissue Biopsy

Fat biopsies were performed on patients recruited at the NMVAHCS at baseline and after 6 months of T therapy. Adipose tissue samples were obtained from the abdominal subcutaneous adipose tissue depot. 2% lidocaine was used to anesthetize the periumbilical area before proceeding with a small incision. Adipose tissue (~500 mg) was collected using a liposuction needle under sterile conditions. Samples were then washed in isotonic NaCl, snap-frozen in liquid nitrogen and kept at −80 °C until utilization for the gene expression study. Pressure was applied on the biopsied area until bleeding stopped completely. The wound was then covered with sterile dressing and subjects were instructed to report any signs of infection, tenderness, redness or a hematoma. One week after the procedure, subjects came back to our research laboratory for inspection of the biopsy site. 

### 2.6. Gene Expression 

*ESR1* gene (NC_000006.12) expression was performed by RT-qPCR at baseline and 6 months. Subcutaneous adipose tissue samples were used for RNA extraction by (RNeasy Lipid Tissue, Quigen #74804). Two-hundred ng of RNA were used for retrotranscription into cDNA, which was performed using SuperScript VILO Master Mix (Invitrogen, Carlsbad, CA, USA) following protocol instructions. FAM labelled TaqMan Gene expression assays (Applied Biosystem, College Station, TX, USA) for *ESR1* (Assay ID: Hs01046816_m1), VIC labelled TaqMan gene expression assay for housekeeping *18S* (Assay ID: Hs03003631) and TaqMan Universal Master Mix were used following the manufacturer’s protocol. Relative quantification (CT gene expression at 6 months vs gene expression at baseline adjusted for housekeeping gene expression at both time points) and data analysis were performed using Real Time PCR system QuantStudio5 and QuantStudio Design & Analysis Software v1.3.1, respectively. 

### 2.7. Statistical Analysis

The study population was divided into 4 categories based on baseline E2 levels (pg/mL): (1) <10; (2) 10.0–15.9; (3) 16.0–19.9; (4) ≥20.0 (first analysis) to establish the impact of different E2 levels on body composition, subcutaneous adipose tissue’s *ESR1* expression at baseline, and in response to T therapy. As reported in Section 2.1, this is a secondary analysis of a study investigating the pharmacogenetics of response to testosterone therapy among HG men [11,12]. Original sample power calculation was performed in order to detect significant differences in the primary outcome of the main project. For the present secondary study, power calculations were performed for detection of differences in main outcome (body fat) between E2-groups. The sample size in E2-Group 1 was small; however, it was adequate to detect differences of 3.9% and 4.2% in % truncal and % total fat respectively, between groups 1 and 2 with a post-hoc power of 80% and *alpha* = 0.05. We used 10 pg/mL as the cut-off for hypoestrogenemia in men for the following reasons: (1) Finkelstein and colleagues reported that 10.0 (pg/mL) is the cut-off of E2 levels below which bone loss occurs in men [15] and (2) the reference values of estradiol in men in the Mayo Clinic Laboratories is 10–40 (pg/mL). We believe that 10.0 (pg/mL) is the cut-off of estradiol below which effects of the oestrogen deficiency could be appreciated in men with low testosterone. Accordingly, the lowest E2 category chosen for this study corresponds to <10.0 (pg/mL). Cut-off of estradiol levels for group 2, 3 and 4 where chosen in order to equalize as far as possible sample size across the three groups while providing simple cut-off for potential application in clinical practice (e.g., 16.0 instead of 16.357). Between groups differences in age were assessed by analysis of variance (ANOVA), while differences in BMI, hormonal levels, *ESR1* expression and body composition at baseline and in response to T therapy were analysed by analysis of covariance (ANCOVA) adjusting for age. To establish the impact of BMI on *ESR1* expression, we also divided our population into 2 groups based on their baseline BMI: (1) <31 and (2) ≥31. A BMI cut-off of 31 was chosen in order to equalize the sample size in the 2 groups analysed. Groups were compared by ANCOVA, adjusting for age and lean mass at first and then adjusting for E2 levels to distinguish between effects mediated by high BMI and obesity-induced hyperestrogenemia. When the overall *p* value was <0.05, posthoc analysis to compare means was performed using Fisher’s least significant difference (LSD) procedure. Simple correlations between variables were tested using Pearson correlation analysis. Data are presented as means ± Standard Deviation (SD) in the tables and text and means ± Standard Error (SE) in the figures. Data was managed using Excel 2013 (Microsoft, Redmond, WA, USA) and analysed using Statgraphics X64 (Statgraphics Technologies, The Plains, VA, USA). A *p* < 0.05 was considered statistically significant. 

## 3. Results

One-hundred and five subjects were initially enrolled, with 90 completing the 6 months assessment. Subcutaneous fat biopsies were performed on 22 and 28 men at baseline and 6 months, respectively. 

Participants baseline mean age was 59.6 ± 8.4 years; average T level by LC/MS was 270.5 ± 84.0 (68.0–481.0) (ng/dL); mean BMI was 32.3 ± 5.5 (range: 21.8–48.6) (kg/m^2^) and mean E2 was 17.0 ± 6.1 (7.8–37.0) (pg/mL). Baseline population characteristics are reported in Table 1. Group 2 (E2:10.0–15.9 pg/mL) was significantly younger compared to groups 3 and 4. Group 2 had the lowest body weight (kg) compared to groups 3 and 4 and the lowest BMI compared to group 4 (Table 1). T levels were lower in group 1 compared to groups 2, 3 and 4 and in group 3 compared to group 4 (Table 1).

**Figure 1:**
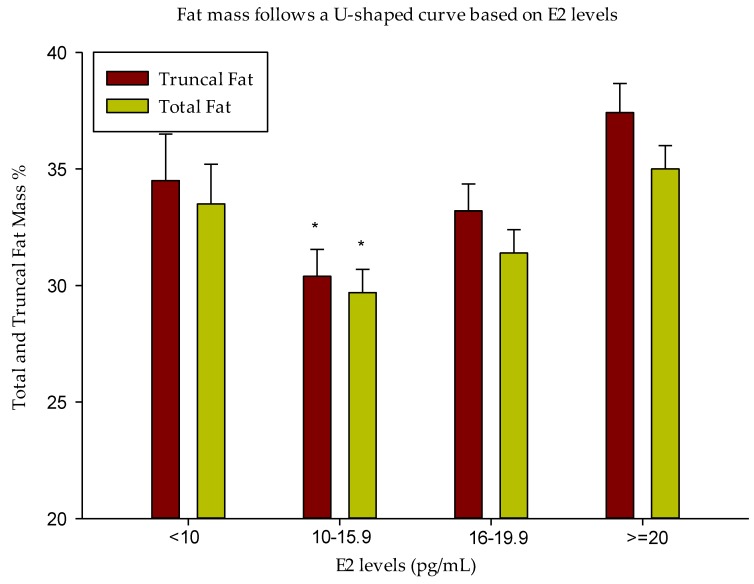
Fat Mass Follows a U-shaped curve based on E2 levels. Total and truncal fat mass (%) were the lowest in group 2. Data are presented as Mean ± Standard Error (SE). * *p* < 0.05 compared to all other groups.

At baseline, total and truncal fat mass (kg and %) followed a U-shaped curve based on E2 levels: they were the lowest in group 2 and highest in groups 1 and 4, that is, at the opposite ends of the E2 spectrum (Table 2 and Figure 1). In addition, % lean mass was higher in group 2 compared to groups 1 and 4, while absolute lean mass (in kg) was highest in groups 3 and 4 (Table 2). These data show that E2 levels between 10.0 and 15.9 (pg/mL) correspond to the best body composition profile in men with low T. 

**Figure 2:**
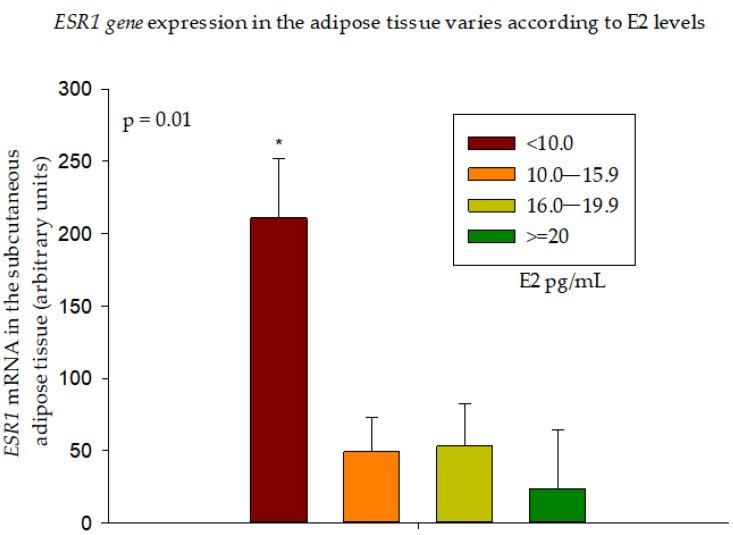
*ESR1* gene expression in the adipose tissue varies according to E2 levels. Group 1 (E2 < 10.0) had the highest *ESR1* expression compared to all other groups (* *p* < 0.05). Data are presented as Mean ± SE.

*ESR1* expression in the subcutaneous adipose tissue was the highest in group 1 (Figure 2), suggesting a higher sensitivity to E2 at the lower end of the E2 spectrum. 

After 6 months of T therapy, circulating T was similar between groups, whereas serum E2 was higher in group 4 compared to group 2 (Table 3). E2 and T increased more (% changes) in group 1 compared to all the other groups, although absolute changes did not differ between groups (Table 3). Body weight at 6 months remained significantly higher in group 4 compared to groups 1 and 2 and in group 3 compared to group 2, whereas body weight changes (kg and %) were not different between groups (Table 3). Similarly, BMI at 6 months was higher in group 4 compared to group 2, although BMI changes did not differ between groups.

There was no significant correlation between 6 months serum E2 and *ESR1* expression (*r* = 0.04, *p* = 0.82) or changes in *ESR1* expression (*r* = −0.18, *p* = 0.55). 

**Figure 3:**
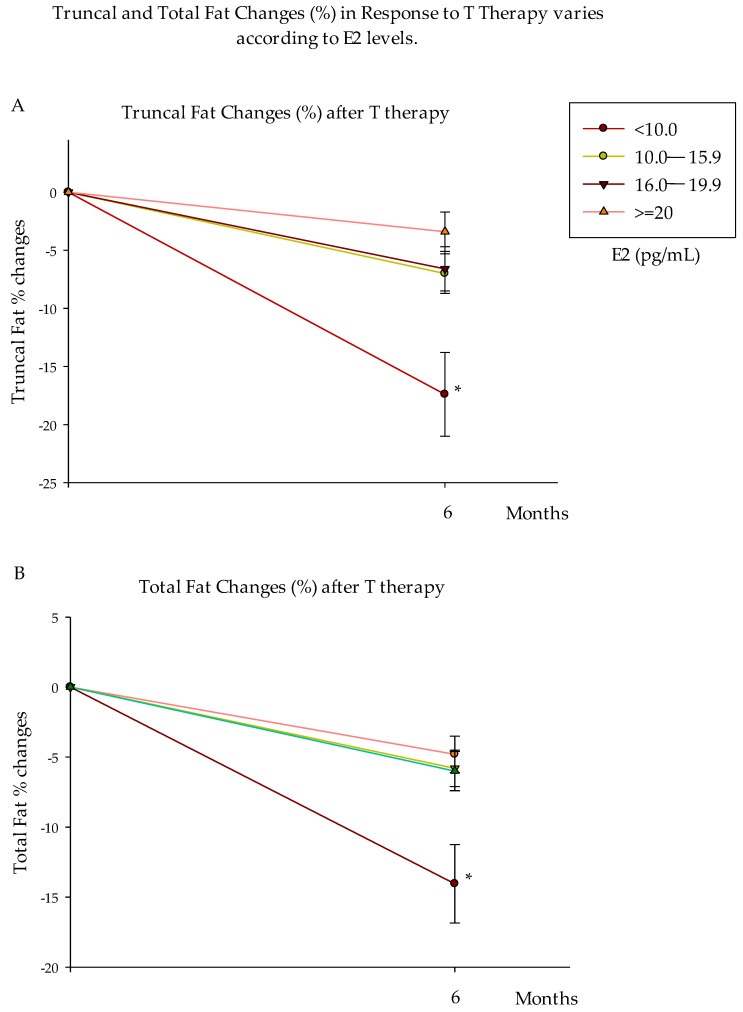
Truncal and Total Fat Changes (%) in Response to T Therapy varies according to E2 levels. Truncal (**A**) and total (**B**) fat mass decreased more (% changes) in group 1 (E2 < 10.0 pg/mL) compared to all other groups. Data are presented as Mean ± SE. * *p* < 0.05 compared to all other groups.

Remarkably, group 1 experienced the highest total (% from baseline) and truncal (kg and % from baseline) fat mass loss compared to all other groups (Figure 3 and Table 4). After 6 months of T therapy, there were no differences in changes in lean mass between groups (Table 4). These data demonstrate that the group with the lowest E2 level loses more fat mass after 6 months of T therapy, possibly because of higher sensitivity to E2 as suggested by the higher *ESR1* expression (mRNA) at baseline.

**Figure 4:**
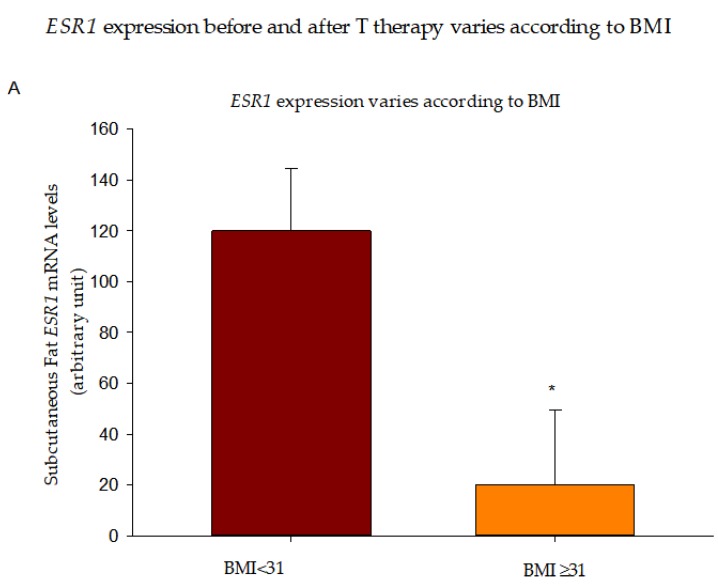

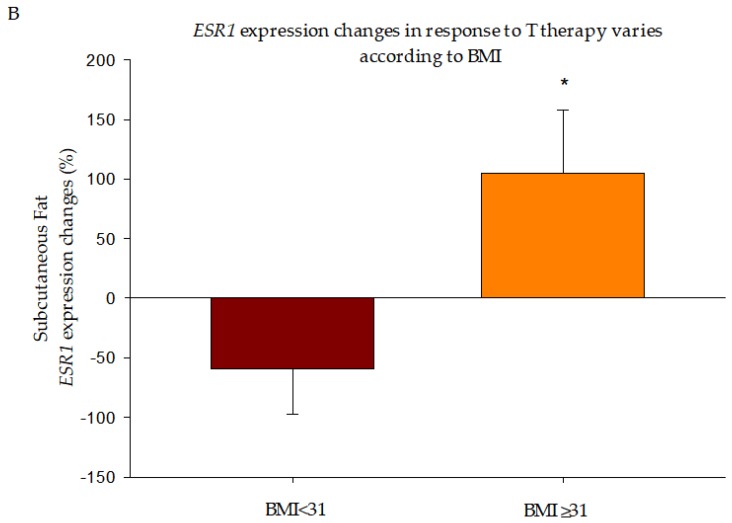
*ESR1* expression before and after T therapy varies according to BMI. *ESR1* mRNA levels were lower in BMI ≥ 31 (**A**) but increased more in this group after 6 months of T therapy (**B**). Data are presented as Mean ± SE. * *p* < 0.05 compared to BMI < 31.

In addition, we divided our population into two groups basing on their BMI and observed that individuals with BMI ≥ 31 (*n* = 9/22) had significantly lower *ESR1* expression at baseline compared to those with BMI of <31 (Figure 4A). This significance was lost after adjusting for baseline serum E2, suggesting that the differences in *ESR1* expression between the 2 BMI groups is dependent on circulating E2 levels. Interestingly, 6 months of T therapy promoted an increase in *ESR1* expression in individuals with BMI ≥ 31 (*n* = 4) compared to the reduced expression in those with a BMI < 31 (*n* = 8) (Figure 4B), which resulted in similar *ESR1* mRNA in the two groups at this time point (data not shown). In this case, significance was maintained after adjustment for baseline E2 but not when adjusting for the E2 levels at 6 months, suggesting that the observed difference was dependent on circulating E2 at 6 months.

## 4. Discussion

Our study shows that HG men with serum E2 between 10.0–15.9 (pg/mL) have the best body composition profile as evidenced by having the lowest absolute and percent truncal and total fat mass and the highest percent lean mass. However, HG men with the lowest baseline E2 (group 1) are the best responders to T therapy, having the greatest reduction in total and truncal fat compared to all other groups. Analysis of subcutaneous adipose tissue showed that *ESR1* mRNA was highest in group 1 at baseline. Taken together, these findings demonstrate that HG men with E2 levels less than 10 (pg/mL) may have the best lipolytic response possibly because of higher E2 sensitivity. Furthermore, we found that men with higher BMI (i.e., BMI ≥ 31) had lower *ESR1* expression compared to their leaner counterparts in an E2 dependent fashion, highlighting the role of available oestrogen in regulating E2 sensitivity. In summary, our results suggest that serum E2 levels between 10–15.9 (pg/mL) may be the optimum level for maintenance of a better body composition in HG men, while levels outside this range are associated with increased adiposity.

Additionally, oestrogen sensitivity (indicated by *ESR1* expression) in HG men is influenced by available oestrogen and those with obesity-induced hyperestrogenemia have reduced E2 sensitivity relative to HG men with lower E2, resulting in a variable response to T therapy.

According to the Center for Disease Control and Prevention, over 39.5% of US adults are obese, a percentage expected to increase in the coming years considering the rising prevalence of obesity among children and adolescents [1]. Although the aetiology of obesity is multifactorial, several hormones play a key role in its induction and progression. Oestrogens are well known regulators of fat distribution and deposition [3]. Their reduction is associated with changes in body composition [4] and a 3-fold increase in the risk of obesity and metabolic syndrome in the postmenopausal period [16]. 

Although testosterone therapy in HG men is associated with reduction in body fat [17,18], investigators from a recent study have shown that oestrogen is the main sex hormone regulating fat mass in men [7]. In an elegant study of Finkelstein et al., men given GnRH agonist (to inhibit endogenous sex hormones production) in combination with increasing doses of T plus an aromatase inhibitor (GnRH agonist + T + aromatase inhibitor) had increased fat mass compared to men who were on GnRH agonist in combination with increasing doses of T plus placebo (GnRH agonist + T + placebo) [7]. Moreover, male to female transgender patients are reported to experience a shift from an android to a gynoid fat habitus after 12 months of E2 and antiandrogen therapy [19]. The coexistence of high visceral fat and elevated E2 in obesity therefore may appear inconsistent. Aromatase, which converts androgens into oestrogens, is highly expressed in adipocytes of obese subjects and is primarily responsible for obesity-induced hyperestrogenemia [20]. We hypothesize that adipocytes of obese hyperestrogenemic subjects display lower sensitivity to the lipolytic effects of E2 compared to individuals with lower circulating E2. In support of this concept, is our result showing that men in the lowest baseline estradiol group had the highest *ESR1* expression. In addition, individuals with a BMI ≥ 31 also exhibit lower *ESR1* mRNA. However, the significance was lost when analysis was adjusted for circulating E2, suggesting that hyperestrogenemia following obesity and not obesity itself, causes lower *ESR1* expression. Nilsson and colleagues showed lower *ESR1* expression in adipocytes of obese compared to non-obese premenopausal women, while reporting increased *ESR1* mRNA levels in those who underwent weight loss from gastric-bypass surgery [21]. However, these authors were unable to conclude whether reduced *ESR1* expression was a cause or a consequence of obesity and whether differences in circulating E2 (not assessed in this study) played a role. Since fat mass follows a U-shaped distribution based on E2 levels in our HG men, the comparably high fat mass and BMI in people with low and high serum E2 suggest that perhaps oestrogen levels and not BMI, influence E2 sensitivity. 

Our study also indicated that individuals with lower E2 (and higher adipocyte *ESR1* mRNA) lose more fat mass after 6 months of T therapy. After T treatment, the groups had similar T and E2 in circulation (except for group 4 who had higher E2 compared to group 2) and experienced similar absolute increase in both hormones. For this reason, we believe that the higher fat mass loss experienced by group 1 is not due to varying absolute amounts of serum T or E2 but instead to a different sensitivity to E2 as indicated by *ESR1* expression. Interestingly, T therapy increased *ESR1* mRNA expression in subjects with BMI ≥ 31 but a reduction in those with BMI of <31, leading to the lack of difference in the receptor expression between the two groups at 6 months. This finding, in conjunction with the loss of difference in baseline *ESR1* expression between the 2 BMI categories after adjusting for circulating E2, underscores the role played by E2 in variable *ESR1* expression. Alternatively, our finding may be due not only to the observed change in *ESR1* gene expression but also to the binding of higher concentrations of E2 to another receptor with a lower binding affinity that could potentially inhibit the response of *ESR1*. Our study has limitations. Total sample size and number of patients who underwent fat biopsy are small. Because this study was not designed to investigate the relationship between E2 and body composition, group 1 was composed of fewer participants compared to the other 3 groups (see method section). Another major limitation in this secondary analysis is the lack of information on the level of physical activity and dietary habits of our participants, which could have an impact on the outcome under investigation (i.e., changes in fat mass). This information was not part of the design in the original study, thus, is not available in this secondary analysis. In conclusion, all together, our results suggest the possibility that the persistently high E2 levels associated with obesity leads to *ESR1* downregulation in adipocytes and reduced E2 sensitivity, which in turn results in attenuated fat mass loss in response to T therapy. Oestrogen resistance induced obesity was first described in a man with an inactivating mutation of the *ESR1* [10]. Lack of E2 action predispose to metabolic syndrome, type 2 diabetes and cardiovascular events [6], conditions that affect both genders [8]. The identification of optimum serum E2 associated with the best E2 sensitivity and body composition profile is of clinical relevance, especially considering its influence on the response to hormone therapy as observed in our study. This is the first study showing: (1) the optimum E2 levels associated with better body composition in HG men, (2) the existence of different E2 sensitivity based on circulating E2 and (3) its influence on the response to T therapy. Prospective investigations with a bigger sample size are needed to confirm our observation and to explore consequences of E2 resistance among individuals with hyperestrogenemia.

## Figures and Tables

**Table 1:** Baseline Characteristics according to Estradiol (E2) levels (pg/mL).

E2	<10.0	10.0–15.9	16.0–19.9	≥20.0	*p* Value
*n* (%)	11 (11.2)	32 (32.7)	29 (29.6)	26 (26.5)	
Age	60.2 ± 7.7	56.0 ± 10.2	60.3 ± 6.2 ^γ^	62.3 ± 7.3 ^γ^	0.03
Body Weight (kg)	99.9 ± 9.5	91.3 ± 11.5	101.7 ± 20.0 ^γ^	109.9 ± 17.4 ^γ^	<0.001
BMI (kg/m^2^)	32.1 ± 3.4	30.0 ± 4.0	32.0 ± 6.0 †	35.0 ± 5.9 ^γ^	<0.01
Testosterone (ng/dL)	166.30 ± 48.8 *	277.9 ± 82.6 †	256.8 ± 74.2 †	323.3 ± 63.2	<0.0001
E2 (pg/mL)	8.8 ± 0.62	13.1 ± 1.30	17.3 ± 1.10	25.0 ± 5.31	<0.0001

All data (except for age) are adjusted for age and are expressed as Mean ± Standard Deviation SD. *n:* number of subjects; BMI; Body Mass Index. ^γ^ *p* < 0.05 compared to group 2; † *p* < 0.05 compared to group 4; * *p* < 0.05 compared to all other groups.

**Table 2:** Baseline Body Composition according to Estradiol levels (pg/mL).

E2	<10.0	10.0–15.9	16.0–19.9	≥20.0	*p* Value
*n* (%)	11 (11.2)	32 (32.7)	29 (29.6)	26 (26.5)	
Truncal Fat					
kg	17.6 ± 3.0	13.4 ± 4.3 *	16.9 ± 7.2 ^γ,^†	21.9 ± 7.0	<0.0001
%	34.5 ± 2.7	30.4 ± 6.2 *	32.8 ± 6.8 †	37.4 ± 6.5	<0.01
Total Fat					
kg	33.8 ± 6.4	26.8 ± 7.5 *	32.0 ± 11.6 †	39.3 ± 11.0	<0.001
%	33.5 ± 3.5	29.7 ± 5.5 *	31.4 ± 5.4 †	35.0 ± 5.5	<0.01
Total Lean					
kg	63.8 ± 5.7	59.8 ± 6.2 †	65.0 ± 8.8 ^γ^	68.4 ± 7.4	<0.001
%	63.8 ± 3.3	67.4 ± 5.3 *	65.9 ± 5.1 †	62.5 ± 5.3	<0.01

All data are adjusted for age and are expressed as Mean ± (SD). ^γ^ *p* < 0.05 compared to group 2; † *p* < 0.05 compared to group 4; * *p* < 0.05 compared to all other groups.

**Table 3:** Hormonal Profile and Body Composition Changes after 6 Months of T Therapy.

E2	<10.0	10.0–15.9	16.0–19.9	≥20.0	*p* Value
*n* (%)	5 (7.2)	23 (32.8)	23 (32.8)	19 (27.2)	
E2 (pg/mL)	37.9 ± 17.8	32.1 ± 31.8	43.3 ± 43.3	53.8 ± 54.0 ^γ^	0.03
%	321.7 ± 180.3 *	145.9 ± 171.3	152.1 ± 140.1	118.5 ± 103.2	0.01
∆	36.2 ± 17.5	22.7 ± 19.6	26.2 ± 21.2	22.3 ± 26.2	0.61
T (ng/dL)	696.7 ± 1 19.3	614.1 ± 387.4	597.8 ± 288.0	786.5 ± 360.0	0.26
%	344.7 ± 226.2 *	144.1 ± 217.9	175.6 ± 167.0	153.2 ± 116.2	0.04
∆	508.3 ± 126.8	337.0 ± 379.5	348.0 ± 291.9	472.9 ± 340.8	0.37
Body Weight (kg)	97.4 ± 9.9 †	91.4 ± 11.4	101.8 ± 19.7 ^γ^	109.8 ± 17.2 ^γ^	<0.01
%	−0.73 ± 6.0	0.67 ± 3.2	0.83 ± 3.8	0.09 ± 3.0	0.7
∆	−0.83 ± 6.2	0.01 ± 3.0	0.7 ± 4.0	0.5 ± 3.4	0.73
BMI (kg/m^2^)	31.5 ± 2.8	30.1 ± 4.0	32.4 ± 5.5	35.0 ± 6.0 ^γ^	0.01
%	−0.74 ± 6.0	0.65 ± 3.2	0.82 ± 3.8	0.09 ± 3.0	0.63
∆	−0.30 ± 2.0	0.03 ± 1.0	0.20 ± 1.3	0.16 ± 1.2	0.71

All data are adjusted for age and are expressed as Mean ± SD. ^γ^ *p* < 0.05 compared to group 2; † *p* < 0.05 compared to group 4; * *p* < 0.05 compared to all other groups.

**Table 4:** Body Composition Changes after 6 Months of T Therapy.

E2	<10.0	10.0–15.9	16.0–19.9	≥20.0	*p* Value
*n* (%)	5 (7.2)	23 (32.8)	23 (32.8)	19 (27.2)	
Truncal Fat					
%	−17.4 ± 10.2 *	−7.0 ± 8.1	−6.6 ± 8.2	−3.4 ± 7.4	0.01
∆ (kg)	−3.0 ± 7.6 *	−0.7 ± 3.7	−1.1 ± 1.4	−1.0 ± 3.6	0.01
Total Fat					
%	−14.0 ± 9.0 *	−4.8 ± 6.2	−5.8 ± 6.2	−6.0 ± 5.5	0.03
∆ (kg)	−4.4 ± 14.7	−1.5 ± 6.5	−1.8 ± 2.1	−1.9 ± 6.6	0.07
Total Lean					
%	5.6 ± 6.3	4.7 ± 4.4	5.0 ± 4.7	3.5 ± 4.4	0.63
∆ (kg)	3.6 ± 23.3	2.8 ± 14.9	3.1 ± 2.8	2.4 ± 4.9	0.81

All data are adjusted for age and are expressed as Mean ± SD. * *p* < 0.05 compared to all other groups.
